# A toolkit with nine district types to support municipalities in taking an integrated approach to prevention

**DOI:** 10.1186/s13690-019-0378-5

**Published:** 2019-12-19

**Authors:** Ilse Storm, Nikkie Post, Antonia Verweij, Karlijn Leenaars

**Affiliations:** National Institute for Public Health and the Environment, Centre for Health and Society, PO Box 1, 3720 Bilthoven, BA Netherlands

**Keywords:** Integrated approach, District types, District health profiles, Evidence-based interventions, Neighbourhoods, Prevention

## Abstract

**Background:**

Not only do people differ in their health, so do districts within municipalities. For example, city centres have different characteristics and health issues than villages or post-war neighbourhoods. This is why the Dutch National Institute for Public Health and the Environment has developed a toolkit, ‘Prevention in the district’, based on nine different types of district.

**Methods:**

The aim of the toolkit is to help municipalities implement an integrated approach to prevention by providing tailored, practical information. We therefore looked at the best way to improve the connection between the available knowledge and local needs. Based on data analysis, expert opinion and working sessions with professionals and local policymakers, we developed a toolkit with three related tools.

**Results:**

The following tools were developed: 1) nine district types with their prominent characteristics and 14 themes for prevention (ranging from loneliness to overweight); 2) a data guide containing a set of indicators to assess the district health profile; 3) a prevention guide containing a mix of evidence-based interventions for the 14 themes. The tools are presented in a toolkit (a clickable PDF) to emphasise the fact that they form a coherent whole. The link between data and interventions is considered to be particularly innovative.

**Conclusion:**

The three tools support the improvement of the health and well-being of residents in a district. The first indications are that the toolkit empowers municipalities and lets them work towards an integrated approach. An integrated approach in both district health profiles and district plans could also serve as an example for other countries.

## Background

### Current situation

In many Western countries, the importance of prevention has long been accepted in theory, but the practical implementation is still a challenge [1–3]. The objective of prevention is ensuring that residents stay healthy by promoting or protecting their health. Another aspect of prevention is preventing or detecting diseases at the earliest possible stage [4]. There are many factors that influence health, such as an unhealthy lifestyle, unfavourable residential and living conditions, less access to care or other services and low income [5, 6]. That is why prevention is focusing more and more not just on individuals but also on their surroundings: in other words, an integrated approach [2]. In the Netherlands an integrated approach is often used interchangeably with the term Health in All Policies. Health in All Policies is a broader complementary policy-related strategy with a high potential for contributing to population health [7, 8]. In practice Health is All Policies is an integrated approach characterized by a mix of interventions and measures from various domains (e.g. care and the physical and social living environments) [9] Not only the Netherlands but other countries too, such as Finland, Sweden, Norway and UK, are promoting the implementation of integrated approaches in local practice (e.g. broad vision on health, exerting influence, capacity building) [6, 10].

Municipalities in the Netherlands are key actors when it comes to preventing health problems among residents. In 2015, the Dutch government delegated tasks in the field of public health to the municipalities in order to bring the organisation of prevention and care closer to residents. Responsibilities and resources were transferred from central government to local government. Municipalities are expected to work on a more integrated approach, which means that there should be collaboration between different domains to prevent health problems [11]. Prevention at a local level is encouraged by the central government through the national prevention agreement of 2018 to tackle complex health problems together using an integrated approach (e.g. smoking, overweight and excessive alcohol consumption). Nationwide incentive programmes support this agreement (such as ‘Healthy in the City’ or ‘Get Prevention Started in your Municipality’) [12–14]. Delegated tasks from national to local and national incentive programmes allow municipalities to implement an integrated approach at the district level together with various partners such as policymakers and professionals as well as residents [15, 16].

### Obstacles to the approach

In the past few years, more and more Dutch municipalities have started integrated approaches in districts (often used interchangeably with the term neighbourhoods) to improve or protect health. However, implementation in practice has come up against a few obstacles [17, 18].

**Fig. 1:**
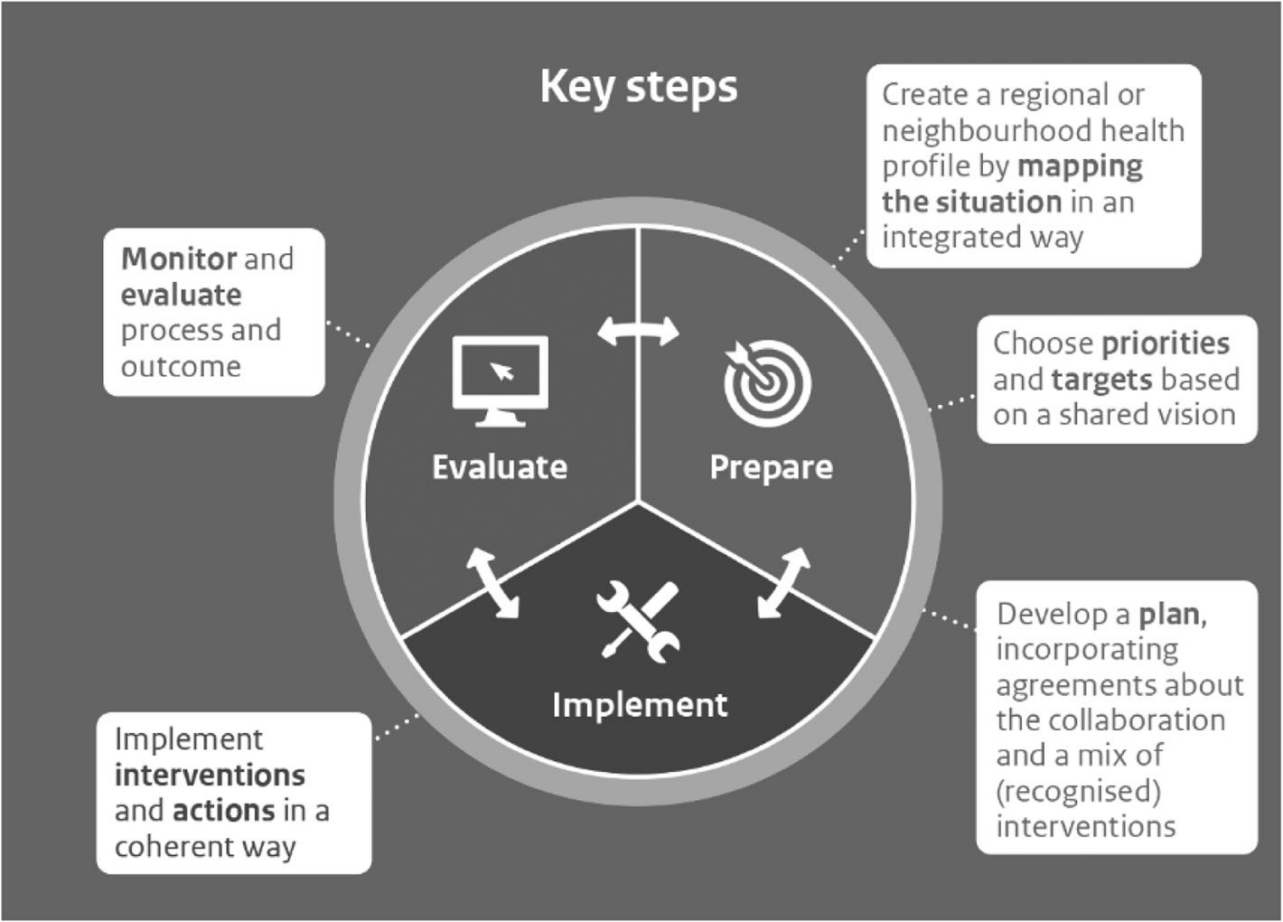
Process steps in an approach for a healthy district

Firstly, it is a challenge for municipalities to implement a preventative approach integrally in a district (see Fig. 1). For instance, data is often only collected about the health of residents and not about their living conditions, or it may not be possible to set up a coherent approach involving interventions from various domains [19, 20]. To develop an effective approach at the district level, municipalities must integrally identify district characteristics together with partners and residents (a district profile for lifestyle and health, levels of amenities, the social and physical environment, participation and demographics) and interpret this so that prioritised themes and an integrated plan can be produced, followed by the execution and evaluation of this approach [21].

Secondly, it is a challenge for municipalities to set up an approach that suits the context when various different districts may require different approaches to health problems [21]. For example, city centres have different characteristics and health issues than villages or post-war neighbourhoods. This means that a different approach is needed [22]. In the past few years in the Netherlands, a great deal of attention has been paid to the disadvantaged districts of large cities [14]. However, there are also other types of districts with relevant health themes that can be tackled locally. In the Netherlands, different types of districts are geographically spread across the country and every municipality has multiple types of districts [23].

Thirdly, disseminating knowledge and putting it into practice locally is a challenge. A lot of knowledge is available nationwide, both about data covering various domains at the district level and about proven, recognised interventions that can assist in an integrated district approach. This knowledge however, isn’t always coherently delivered in a way that the process of promoting district profiles to healthy district approaches is stimulated. An integral overview in which the available data about the health issues and characteristics for certain types of district is combined with proven, recognized interventions can provide support in the implementation of an integrated working approach in practice [24, 25]. Studies revealed that policy advisors need guidance in their search for and selection of information [26, 27].

In view of these obstacles to implementing of an integrated approach to prevention, the Dutch National Institute for Public Health and the Environment (RIVM) developed a toolkit called ‘Prevention in the district’. Hereby we have been borne on previous (international) studies with regard to tools and use of knowledge for evidence-informed health policy making (such as making decisions based on the best available research and using systematic data and information and development of integrated plan tailored to the local situation) [17, 19, 28].

### Development of the toolkit

The aim of the toolkit is to enhance an integrated approach to prevent health problems. This integral way of thinking could possibly also help other countries in an integrated approach to health issues. The toolkit is not a blueprint but is instead aimed at inspiring municipal policymakers or professionals and providing them with concrete tools about data, themes and interventions to start working on their own district or municipality. Municipalities are enabled to extract available knowledge for their own context. A local process supervisor (e.g. a health policymaker, a health expert or professional community health services) is able to take the initiative to use the toolkit in practice. In order to collaborate successfully between domains the toolkit will not suffice, additional actions are required. For example, the establishment of a process approach and the organization of related networks on neighbourhood level, and more specifically: attention for success factors (e.g. governance, consensus) [29, 30].

Three tools were developed for this toolkit: 1) nine district types with prominent characteristics and 14 themes for prevention; 2) a data guide containing a set of indicators to develop an integrated district health profile; 3) a prevention guide containing a mix of recognised interventions for the 14 themes. The tools are presented together in a toolkit; this emphasises the coherence between the tools and has the advantage of a single location for all three tools. The toolkit was developed by RIVM in cooperation with various partners, commissioned by the parties involved in *Agenda voor de Zorg* [Agenda for Care], the Association of Dutch Municipalities and the Ministry of Health, Welfare and Sport to promote work on prevention [16].

## Methods

Various methods were used to develop the three related tools — district types, a data guide and a prevention guide — for the toolkit (see Table 1). The toolkit was developed during the period from April 2016 to February 2018.

**Table 1:** Methods for developing the district types, data guide and prevention guide

Methods	Implementation	Indicators and networks	Main questions
District types			
Data analysis	Cluster analysis	Domains providing data - health and lifestyle - amenities - physical environment - social environment - demographics	Which district characteristics are associated with one another?
Working sessions	9 meetings averaging 7–8 people	Participants: - area advisor - district nurse - neighbourhood sports coach - GP - advisor from municipal public health services - policy advisor - paediatrician - community worker - advisor from regional primary care support structure	What are the most important characteristics of this type of district? What are the biggest problems or themes that you would like to tackle? What developments can you see?
Expert opinion	Interviews, 6 people	Representatives: - Research firm for the typology of residential environments - Netherlands Institute for Social Research - Netherlands Environmental Assessment Agency - RIVM (3)	To what extent does the overview give a familiar picture? What are familiar aspects, what are surprising aspects and why is that? How could we improve or refine the overview?
Data guide			
Selection of indicators	Desk analyses	Data from six domains: - health and lifestyle - amenities - physical environment - social environment - participation - demographics	Which indicators occur in multiple indicator sets? For which indicators are national sources available at the district level?
Expert opinion	Meetings	Representatives of knowledge institutes: - Public health - Mental health - Healthcare - Health inequalities - Social issues	To what extent are the selected indicators relevant and should they be included? What is still lacking in terms of indicators and sources?
Prevention guide			
Database search and grey literature	Desk research	Information was taken inter alia from: - database with lifestyle interventions - database with interventions for young people - database with social interventions - supplemented by grey literature	What interventions are available for the high-priority themes in the districts? What interventions tie in with the four blocks of the integrated approach?
Expert opinion	Meetings and feedback in writing, 13 people	Representatives of knowledge institutes: - Public health (2) - Mental health - Healthcare - Health inequalities - Social issues (2) - Youth (2) - RIVM (4)	To what extent does the overview of interventions give a familiar picture? What interventions are missing?

### Nine types of district

Three steps were involved in developing the model of the district types, namely (a) data analysis, (b) working sessions and (c) expert consultation.

- *Data analysis*: a cluster analysis was used to determine whether there are associations between specific characteristics of the districts. The analysis included indicators and characteristics from six domains: health and lifestyle, amenities, the physical environment, the social environment, participation, and demographics. The analysis showed that urbanisation was a particularly important factor determining the different outcomes (e.g. perceived health). The Netherlands already has a classification based on urbanisation, namely the typology of residential environments developed by a research firm, so this was used as the basis [23]. This typology comprises 13 residential environments. Characteristics covering inter alia the health domain (e.g. smoking, overweight, perceived health) and the social domain (e.g. providing informal care) were then added for all the districts. The characteristics were distinctive for nine of the 13 residential environments. Some residential environments were merged because there was not enough of a difference between the characteristics (e.g. ‘urban centre’ and ‘urban centre plus’ were merged). This resulted in nine district types.
- *Working sessions with local stakeholders*: a working session was held for each district type with stakeholders working in that type of district. The aim of the session was to obtain additional information based on their experiences. A total of nine working sessions were held. For the sessions, we first looked in our own network for stakeholders whom we could invite. This list of stakeholders was supplemented using the snowball sampling method with the aim of ensuring representation among the stakeholders of as many domains as possible (prevention, healthcare, physical environment and social environment). In the meetings, they discussed the key characteristics of each type of area, the main health problems and the kinds of developments that are being seen.

The figures produced by the data analyses per district type were also discussed at the end of the working sessions. The results of the data analyses and the working sessions were summarised for the nine district types, in the form of a district story and an overview of the district characteristics divided into the six domains of health and lifestyle, amenities, physical environment, social environment, participation, and demographics.

c. *Expert opinions*: an expert round was used to test the results and create further underpinning for the characteristics of the district types. Social geographers and other experts were approached for this purpose. The experts were interviewed after having been sent the overview of district types (characteristics and themes) beforehand.

The results of the working sessions, the quantitative evidence and the expert assessments were used to identify one or two health themes for each of the nine district types that could then be tackled in prevention activities. A total of 14 themes were identified that could be dealt with in the public health domain and related domains.

### Data guide

In addition to the district types, a data guide was also included in the toolkit. The data guide contains indicators and the associated data sources. The data guide gives indicators for which figures from national sources are available per district. Having national data makes it possible to compare districts against the national average or make comparisons with another major city as a reference. Indicators that already appear in multiple national sets were used to ensure a link with existing indicator sets [31–34]. RIVM has calculated figures for all districts in the Netherlands for a number of indicators [24]. If no national sources were available for certain relevant indicators, examples of regional or local sources were given. The selection of indicators listed in the data guide was discussed with experts working at national knowledge institutes in public health, mental health, healthcare, health inequality and social issues.

### Prevention guide

The toolkit also includes a prevention guide that works out the details of an approach for tackling 14 prevention themes. These are the themes that stand out most in the nine district types (based on the data, the working sessions and the expert opinions). The detailed approach was developed using various databases with recognised interventions, e.g. a database with lifestyle interventions, a database with youth care interventions and a database with welfare interventions. Grey literature was also used in addition to these databases [35].

The available databases operate with a formal recognition system. That means that, *the Netherlands has various databases that provide access to information on the quality, effectiveness and feasibility of a wide range of interventions. The information is collected and assessed systematically and the interventions are classified as ‘well described’, ‘theoretically sound’ or ‘effective’. This recognition system for interventions was developed in collaboration with national centres of expertise outside the health promotion sector: social affairs and welfare, child and youth services, long-term care, mental health services and sports. All the partners agreed to use the same assessment process and to encourage the uptake of interventions in their own field. A condition to be acknowledged is that the intervention must be executed in the Netherlands. Also foreign interventions can be acknowledged, provided that they are executed in the Netherlands. This nationwide assessment approach is aimed at promoting integrated policy and practice across the Netherlands*.

An integrated approach is shown for the 14 prevention themes, as using a combination of recognised interventions has more effect that implementing a single intervention [25]. This integrated approach consists of four blocks: 1) education and information; 2) alerting, advice and support; 3) the physical and social environment; and 4) regulations and enforcement [35, 36]. The integrated approach for these themes is tailored to suit the characteristics of the district type in question. Using the intervention databases, we looked for available, recognised interventions that could be linked to the prevention themes that had been prioritised for each district. If no recognised interventions were available, we used the grey literature to look for relevant measures or interventions. The set of interventions and measures was assessed and supplemented by national science institutes (in the same way as for the data guide). See Table 1.

## Results

**Fig. 2:**
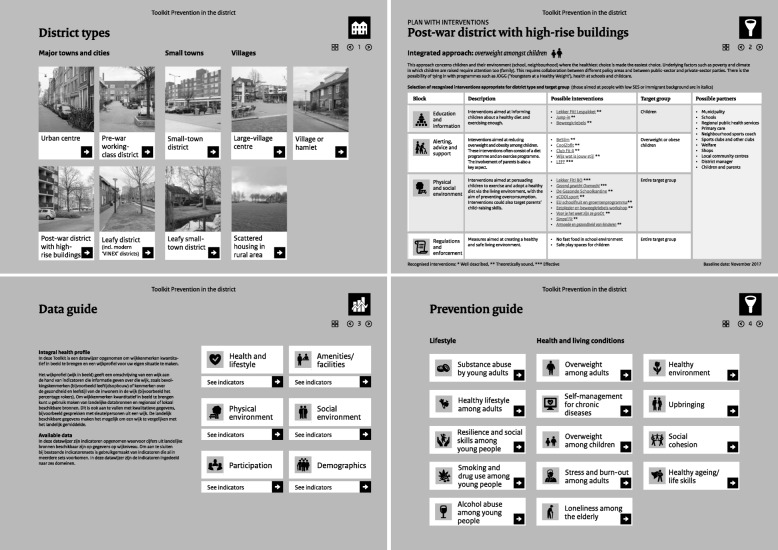
The three related tools in the clickable toolkit and an example district plan

Three related tools were developed for the ‘Prevention in the district’ toolkit, namely district types, a data guide and a prevention guide. They are described below. Figure 2 shows a number of home pages for the clickable toolkit, which can be found at https://www.rivm.nl/gezonde-wijk/preventie-in-wijk/toolkit [16].

### Nine district types

Nine district types are presented in the toolkit. Each district type starts with a ‘district story’, giving a description of the most notable characteristics of the district in the six domains of health and lifestyle, amenities, the physical environment, the social environment, participation, and demographics. Suggestions are also made as to which indicators in the accompanying data guide should be given attention for each district type. Furthermore, one or two high-priority themes are specified for each district type, for which an appropriate integrated approach can be found in the prevention guide. Table 2 gives an overview of the nine district types that have been developed and associated prevention themes.

**Table 2:** Overview of nine district types with the associated characteristics, themes and approach

District types	Data guide^a^	Prevention guide^b^	
	*Key characteristics*	*Selected themes*	*Integrated approach*
Urban centre	**Stimulants, stress, loneliness**, fewer chronic diseases, sufficient exercise and sufficiently healthy diet High level of amenities (shops and public transport), healthcare facilities High-density housing, **poor air quality and little greenery** Low level of social cohesion, and nuisance in the street. Lots of people in work Lots of single-person households and diversity	High level of stimulant use among young adults (with high socioeconomic status) Healthy living environment	Interventions focused on information via peer-group education (‘Unity’), offering self-help programmes (Jellinek online self-help), a policy of enforcement by limiting the supply of stimulants, enforcement of age limits and training cafe staff (‘smartconnection’). Interventions aimed at better utilisation of green areas and green connecting passages, thereby giving more opportunity for people to relax, meet up and exercise (cycling scores well), management and maintenance of greenery and include greenery in spatial plans.
Pre-war working-class district	**Chronic diseases,** mental health issues, **unhealthy patterns of diet and exercise, and poor self-management** High level of amenities (shops and public transport), and **primary care facilities** (GPs) High housing density, little room for exercise and a lot of social housing High degree of social cohesion Fewer initiatives by the public Mixed age distribution, diversity and preponderance of low-income groups	Unhealthy lifestyle among adults with low socioeconomic status Self-management for chronic diseases	Interventions that focus on information about healthy lifestyles (buying and cooking healthy food), healthy lifestyle assistance (‘SLIMMER’) and creating a healthy environment (‘45+ football’) in which healthy behaviour is encouraged. Interventions aimed at enhancing easy-to-understand information about diseases, encouraging people to get a grip on their health and be in control (‘Social and Vibrant’, ‘Exercise Course’), meeting fellow sufferers/healthcare providers online or mobilising the social network (community support).
Post-war district with high-rise buildings	**Unhealthy lifestyle, overweight, mental health issues**, loneliness, stress High level of amenities (shops and public transport), and welfare facilities Unattractive greenery and renovation of homes Sense of insecurity, initiatives from the public Fewer people in work Diversity, a lot of young people, more low-income groups	Overweight among children Climate in which children are raised	Interventions that focus on information about a healthy diet and exercising enough (‘Nice and Fit’), assistance for children and their families in losing weight (‘Lifestyle’, ‘Energy Fun and Friends’) and creating a healthy environment (‘sCOOLsport’, ‘Healthy Weight Overvecht’ and keeping fast-food chains out). Interventions aimed at improving parents’ child-raising skills (‘Child-raising & So On’, ‘Triple P’), improving family members’ skills, better provision of healthcare and facilities for children and young people with behavioural problems (‘the Peaceful School’) and creating a safe environment.
Leafy urban districts	**Stress, mental health issues and excessive alcohol consumption** Few healthcare and welfare facilities in the vicinity Sufficient greenery and space, investing in your own home **Providing informal care** Dual-income couples/people in work Mixed age distribution, more graduates and elderly people depending on age of housing	Stress and burn-out among adults	Interventions aimed at enhancing mental well-being and reducing mental-health complaints. This could involve online self-help programmes or group courses (‘Psyfit.nl’ or ‘Living in Full’). Furthermore, creating an environment where there is room to rest and relax (business yoga).
Small-town districts	**Stimulants (smoking, drugs)**, mental health issues, loneliness and unhealthy lifestyle Basic amenities (shops and public transport), adequate primary care and welfare facilities **Shortage of appropriate housing and unattractive surroundings for exercise** **Nuisance (loitering youngsters) in some places** Lots of people in work Mixed age distribution, diversity, both high-income and low-income groups	Resilience and social skills of young people Smoking and drugs among young people	Interventions aimed at enhancing young people’s social skills (‘Power in Control’) as a way of preventing problem behaviour later on, as well as interventions aimed at giving young people exhibiting problem behaviour new skills (‘Down to Work’, ‘Star Training’). Also creating an environment in which young people can grow up resilient and with social skills (school-wide positive behaviour support) and where nuisance caused by youngsters is tackled (bans on assembly, area bans). Interventions aimed at making young people aware of the risks of smoking and drug use, helping them to stop (‘Pot Check’, ‘Smoke Alert’) and creating a no-smoking environment (ban on smoking at schools and sports locations).
Leafy small-town districts	**Loneliness, not enough exercise,** fewer chronic diseases Adequate amenities (shops, schools) but no healthcare facilities nearby Sufficient greenery and space Little nuisance but **little engagement** Providing informal care Mixed age distribution, diversity and **more one-person households**	Social cohesion	Interventions aimed at flagging up vulnerable inhabitants in danger of becoming lonely (‘Social and Vibrant’, ‘Fancy a Friendship’) and interventions geared to meeting people (‘Hearty Resto’, local sports clubs).
Large-village centres	**Excessive alcohol consumption, overweight and mental health issues** **Declining number of amenities (sports and public transport)**, adequate primary care facilities Sufficient space and greenery, few high-rise buildings High degree of social cohesion and rich ecosystem of societies Lots of people in work Mixed age distribution, more people with few qualifications	Excessive alcohol consumption among young people	Interventions aimed at providing information about alcohol consumption (‘PAS’), assisting young people with alcohol problems and their parents (‘Moti-4’), creating a social norm for responsible alcohol consumption (‘Succeeding Together’, ‘Regional Training Centre Plan of Attack’) and measures aimed at discouragement and enforcement.
Villages and hamlets	**Excessive alcohol consumption**, less exercise (dependent on the car) and **more overweight** **Low level of amenities**, and secondary care some distance away Poorer air quality, a lot of space and greenery High degree of social cohesion and dependency due to ageing (ability to cope) **Providing informal care** Both high-income and low-income groups, fewer young people and more people with relatively little education	Healthy and old/ability to cope	Interventions aimed at creating awareness about ageing healthily and at promoting a healthy lifestyle (functional training for the elderly, ‘Groningen Active Living Model’). Furthermore, interventions aimed support for informal caregivers, contact with fellow sufferers and mobilising the social network around the elderly (centres where they can meet up, community support).
Scattered housing in rural areas	**Less exercise, overweight, loneliness** and stress. **Few amenities**, limited public transport and limited fibre-optic cabling A lot of space and greenery Coping together and rich ecosystem of societies Few employment opportunities Both high-income and low-income groups, fewer young people	Loneliness among the elderly Overweight in adults	Intervention aimed at increasing awareness (‘Week Against Loneliness’) and expanding the social network of vulnerable elderly people (‘Welfare on Prescription’, ‘Social and Vibrant’). Furthermore, interventions aimed at the environment and centred on meeting people (local sports clubs, ‘Hearty Resto’). Interventions aimed at providing information to people at risk of becoming overweight about healthy diets and exercise behaviour (cheap healthy food), improving skills related to healthy diet and healthy exercise (‘Smartsize’, ‘Step into Health’) and encouraging a healthy living environment (‘45+ football’ and offering swimming activities).

^a^Data guide with notable characteristics based on the six domains: 1) health and lifestyle, 2) amenities, 3) social environment, 4) physical environment, 5) participation, 6) demographics (characteristics in bold are the relevant indicators to be considered)

^b^Prevention guide with approach for the high-priority themes based on four blocks: 1) information and education, 2) alerting, advice and support, 3) social and physical environment, 4) regulations (one or two themes are prioritised per district for an integral approach)

### Data guide

The subdivision into six domains was used again for the indicators in the data guide. A reference to the relevant data source is included for each indicator. The indicators from the national sources have been supplemented using data from registers and questionnaires. Some examples of the first category of data are the proportion of insured people with type 2 diabetes, and the level of safety in the district. The data from the questionnaire concerns for example the proportion of people who feel they are in good health and the proportion of people who have provided informal care. Some examples of indicators from regional and local sources are the concentration of nitrogen dioxide and the proportion of people who feel that there are sufficient facilities available for the elderly. When drawing up an integrated district profile, this quantitative data can be supplemented with qualitative data, for example based on talks with key individuals in a district.

### Prevention guide

In the prevention guide an integrated approach has been developed for 14 prevention themes. See also Table 2. It is important to embed an integrated approach in a broader policy strategy [2]. That is why the prevention guide starts with a brief sketch of the policy strategy for each theme, such as the policy domains that should be involved and the local programmes that links could be made with. For the integrated approach, suggestions are given for recognised interventions in each block and it is possible to click through to more information about the interventions. The prevention guide is tailored to suit the characteristics of the district type in question. That means, for example, that in the case of post-war neigbhourhoods with high-rise buildings and a lot of people with low socioeconomic status (SES), most of the interventions listed have been developed specifically for this target group. This means the available data is linked to recognised interventions that could be deployed by the municipality. See Fig. 2 as well for the example of post-war districts. In the toolkit most of the available recognized interventions from databases are for block 1 (education and information) and 2 (alerting, advice and support). Less recognized interventions are available for block 3 (physical and social environment) and 4 (regulations and enforcement). Any proven, recognized interventions out of these domains (that will get available in the future), could be added and made available through a new version of the toolkit.

## Discussion

Local authorities can play an influential role in the health and welfare of their residents [1]. To provide policy workers and professionals with support in doing this, a toolkit — ‘Prevention in the district’ — has been developed with nine different district types, an associated data guide with six domains and a prevention guide covering 14 themes about lifestyle, health and living conditions [16]. The toolkit offers opportunities but it also has some limitations.

### Toolkit’s potential and limitations

The toolkit offers opportunities and concrete tips for designing an integrated approach to prevention for any given district in the Netherlands. The district types described in this toolkit constitute a good starting point for municipalities for a dialogue with partners and local residents from an integral perspective (e.g. covering prevention, the physical environment, the social environment and healthcare). Using the data guide and prevention guide, the district types can also be used to fill in the details of the process from integrated district profile to integrated district plan. In working with district types, the toolkit also shows that not every district faces the same health problems. Different problems dominate in town and city centres compared with villages, such as the more prevalent unhealthy lifestyles in pre-war working-class neighbourhoods, elderly people’s ability to cope, which is under pressure more in hamlets and villages than elsewhere, or the greater prevalence of smoking among young people in small-town districts. That requires a specific approach that fits with the characteristics of the district. The toolkit combines the available knowledge on both the data and the interventions. These links in particular are considered to be innovative and they offer more options for tailored solutions. Delivering more customised solutions at the local level also ties in well with the process of devolving tasks to lower tiers in order to bring the organisation of prevention and healthcare closer to residents and make it more effective [37].

The toolkit is not intended as a blueprint but as a helpful tool for skilled health promotion policymakers. If an integrated approach is to be developed that suits the local context, municipalities will always have to take steps to set that process up properly and systematically [2, 21]. That requires successful, comprehensive collaboration with policymakers, partners and inhabitants, the collection of both quantitative and qualitative data available at the national, regional and local levels, and fleshing out, based on high-priority themes, an integrated approach that fits with the target group’s wishes and needs. This can, for example, involve discussing the quantitative data with residents or creating broad support among administrators for a prevention theme or funding for an integrated plan [35, 38]. So, although the toolkit provides municipalities with guidance on how to tackle this, policymakers and professionals may still need more support when using the tools in their particular district or municipality, or they may still be looking for more know-how. For example, methods for getting the residents involved or generating broad (financial) support or ownership of the integrated approach.

### Implementation and further development of the toolkit

A year after its initial release an estimated 10% of the 355 Dutch municipalities make use of the toolkit. National programmes encourage the use of the available know-how and tools for developing and implementing local preventive health policies [2]. One example is the implementation programme ‘Get Prevention Started in your Municipality’, in which six regional collaborative ventures are using the ‘Prevention in the district’ toolkit as one of the tools [13]. Such programmes not only raise awareness of the toolkit and help spread and implement it, but also provide information about people’s experiences with using it and the possible need for support among users. In addition to these programmes, use of the toolkit is promoted as part of RIVM’s regular tasks [13].

The initial experiences show that users find sufficient information, guidance and tools in the toolkit but they sometimes need advice and support when putting it into practice in their own local context. They have questions such as what data should be chosen, who should be brought in and when in order to develop their own district profile, and which interventions should be selected. One of the benefits of the toolkit is that it brings together existing knowledge and tools (compared to stand-alone tools). However, it is recommended to evaluate this perceived benefit. It is also important to keep the toolkit up to date based on people’s experiences and to develop it further using new data sources or recognised interventions or measures, including in the social environment, physical environment and healthcare domains. For instance, RIVM is constantly adding to the health-related indicators at the district level while new recognised interventions are available for a number of prevention themes [39, 40]. That means that the toolkit in its current form has to be turned into a dynamic, evolving product to which more and more knowledge about effective prevention can be added.

### Added value of the toolkit from an international perspective

The toolkit is a method for promoting an integrated approach. An integrated approach in both district health profiles and district plans could also serve as an example for other countries. Even collating all the available information on both the data and the recognised interventions in one practical toolkit can be beneficial. There are also developments in other countries aimed at working more with recognised interventions that can be used to realise an effective integrated approach. France and Germany, for example, are considering setting up a recognition system for interventions along the lines of the Dutch model [41]. One caveat is that the specific content of the toolkit cannot be taken on board unaltered. The data is Dutch data and the interventions are Dutch interventions. The toolkit also assumes that public health is set up in such a way that municipalities are responsible for prevention. This may differ per country [42]. However, other countries could benefit from practical tools such as the use of district types. Ever since 2012, the WHO has stressed the importance of practical tools for encouraging collaboration between multiple domains [3, 6].

## Conclusion

Fleshing out integral prevention and putting it into practice, making the transition from knowledge to the specific local situation, is a complex process. The toolkit ‘Prevention in the district’ with three tools offers insights and concrete tips. A locally implemented process with stakeholders and local residents is still needed to tackle the prevention themes and tailor the recognised interventions to suit the local context. There are also opportunities for improving the toolkit by making more sources available nationally with data at the district level, by working out other relevant prevention themes in detail and by including more recognised interventions in the social environment, physical environment and healthcare domains. The first indications are that the toolkit empowers municipalities and lets them work towards an integrated approach. An integrated approach in both district health profiles and district plans could also serve as an example for other countries.

## Data Availability

Please contact first author for data or material requests.

## Abbreviations

GGD GHOR NL: Association of GGD's (Community Health Services) and GHOR (Regional Medical Emergency Preparedness and Planning) offices in the Netherlands
Movisie: National science institute offering a comprehensive approach to social issues
NJi: Netherlands Youth Institute
Pharos: Dutch Centre of Expertise on Health Disparities
RIVM: National Institute for Public Health and the Environment Agenda voor de zorg-partijen: care providers in the Netherlands
ROS: Regional primary care support structure
Trimbos-instituut: Netherlands Institute of Mental Health and Addiction Ineen: Primary care organisation
VNG: Association of Dutch municipalities
VNG: The Association of Dutch Municipalities
VWS: The Ministry of Health, Welfare and Sport
